# A cross-sectional investigation on remote working, loneliness, workplace isolation, well-being and perceived social support in healthcare workers

**DOI:** 10.1192/bjo.2024.7

**Published:** 2024-02-26

**Authors:** Dearbhla O'Hare, Fiona Gaughran, Robert Stewart, Mariana Pinto da Costa

**Affiliations:** Institute of Psychiatry, Psychology and Neuroscience, King's College London, UK; Psychosis Studies, Institute of Psychiatry, Psychology and Neuroscience, King's College London, UK; and National Psychosis Service, South London and Maudsley NHS Foundation Trust, London, UK; South London and Maudsley NHS Foundation Trust, London, UK; and Institute of Psychiatry, Psychology and Neuroscience, King's College London, UK

**Keywords:** COVID-19, remote working, well-being, loneliness, perceived social support

## Abstract

**Background:**

Following the onset of the COVID-19 pandemic, healthcare trusts began to implement remote working arrangements, with little knowledge of their impact on staff well-being.

**Aims:**

To investigate how remote working of healthcare workers during the pandemic may have been associated with stress, productivity and work satisfaction at that time, and associations between loneliness, workplace isolation, perceived social support and well-being.

**Method:**

A questionnaire was developed to explore remote working and productivity, stress and work satisfaction during time spent working remotely. Associations between current loneliness, workplace isolation and well-being, and the influence of perceived social support, were explored with perceived social support as a potential moderator.

**Results:**

A total of 520 participants responded to the study, of whom 112 were men (21.5%) and 406 were women (78.1%), with an age range of 21–77 years (mean 40.0, s.d. = 12.1). Very few (3.1%) worked remotely before the COVID-19 pandemic, and this had increased significantly (96.9%). Those who worked ≥31 h a week remotely reported higher stress and lower workplace satisfaction at that time, compared with office work, yet also felt more productive. Current loneliness, workplace isolation and perceived social support were cross-sectionally associated with lower current well-being.

**Conclusions:**

Those who worked more hours a week remotely during the pandemic reported increased stress, which may be related to the lack of resources in place to support this change in work.

In the UK, a nationwide quarantine was announced on 23 March 2020 in response to the outbreak of the COVID-19 virus. To curb the spread of the virus, precautions such as physical distancing measures were implemented.^1^ This included restrictions in workplaces including healthcare trusts,^2^ with many people required to switch, at least partially, to remote working, delivering health-related services through telephone and video appointments.^3^ Many National Health Service (NHS) healthcare staff were working from home or shielding and contributing remotely, subject to the availability of resources to facilitate this.^4^ Remote working yielded several advantages for the employee and the organisation, particularly in healthcare, including protection from the virus and reduction of the impact on healthcare provision.^5^ However, it has also been associated with changes in stress, productivity and employee work satisfaction. Previous studies also indicate that this type of working is associated with increased experiences of current loneliness and workplace isolation among female academics in the UK.^6^

## Remote working and COVID-19

When remote working was introduced for many during the COVID-19 pandemic, most organisations were not able to provide the employees with the skills and resources required. This led to many employees experiencing increased stress levels during this time compared with previous office work.^7^ Research has also found similar associations between increasing hours spent working remotely with work satisfaction. Working from home for a longer period of time can particularly reduce work satisfaction when the autonomy to do so is lost, as was the case for many during COVID-19.^8^ In contrast, for those who have had to increase the intensity of working from home in the UK since COVID-19, productivity has increased.^9^ During the COVID-19 pandemic, it was not just working arrangements that were affected, there was also a significant disruption to social activities, which contributed to higher levels of loneliness and stress among people in the UK.^10^ However, the time people spend, on average, working in the UK is increasing, as is the time spent working remotely.^11^ This heightens the need for and importance of research in this area for healthcare workers.

## Loneliness, workplace isolation and well-being during COVID-19

Loneliness is a subjective feeling of prolonged emotional distress based on dissonance between perceived and desired social relations.^12^ This includes a perception of lack of social support, as well as feelings of vulnerability in society and inability to cope with external daily life stressors.^13^ Workplace isolation can be described as an individual's perceived isolation from their colleagues and from the organisation's support network.^14^ When working exclusively remotely during the pandemic, social interactions with co-workers were significantly reduced.^15^ When interactions between colleagues occur during virtual meetings, a person's psychological need to belong is not often fulfilled, as the conversations remain work and task focused.^16^ Furthermore, working from home can lead to employees feeling at a disadvantage compared with those working in-office, because of infrequent contact with managers and supervisors.^17^ Recent systematic reviews on the well-being of healthcare workers since the pandemic have reported elevated levels of ill mental health and poor well-being.^18^ As restrictions ease and working returns to a more hybrid structure for many, these increased experiences of poor well-being may remain.

In a USA study, the well-being of those working from home was negatively affected; however, the support that remote workers experienced acted as a protective factor against the harmful effects of loneliness.^19^ Perceived social support refers to how sufficient and available one perceives social support to be, whereas received social support refers to the perceived quantity and quality of the support given.^20^ Perceived social support is relatively stable throughout one's life, and can buffer stress and promote coping during difficult periods of life.^21^ Because of the sudden change in working arrangements for many during the COVID-19 pandemic, organisations and managers were left with little time to sufficiently prepare for the mental health considerations of their employees.^22^ People with high perceived social support may benefit from its moderating effect on the relationship between loneliness and negative well-being.^23^ When people perceive themselves to have high social support accompanied by higher levels of stress, the stress-buffering hypothesis explains that these people will experience less negative outcomes than those who have lower social support.^24^ Perceived social support is therefore thought to act as an important moderator of the stressor-psychological relationship.^25^

## Healthcare workers and the COVID-19 pandemic

Although the COVID-19 pandemic is abating, the impact of remote working and isolation on well-being of healthcare workers in the NHS still needs to be considered, as many clinicians are providing telephone or online consultations, saving on waiting and travelling times. The NHS also plans for every patient in England to be able to avail themselves of this option in the next 5 years.^26^ Loneliness, and the identification of factors that buffer against it, have been highlighted as a priority in mental health research since COVID-19.^27^ Reviews of the literature provide insight on well-being as being important for workplace outcomes in healthcare workers, and should be prioritised in research.^28^ A mixed-methods study on UK mental healthcare highlighted a number of key issues faced by staff, such as adapting quickly to new ways of working, technological difficulties in remote work and challenges providing sufficient support with reduced numbers of face-to-face contacts.^29^ These additional stressors in work could significantly contribute to poorer well-being outcomes of healthcare staff.

The aim of this cross-sectional study was to explore well-being measures alongside healthcare workers’ experiences of remote working in a large mental healthcare provider. Further, we explored cross-sectional associations between loneliness, workplace isolation and well-being of healthcare employees. We also investigated whether there was a moderating effect of perceived social support on the cross-sectional associations of loneliness, workplace isolation and well-being outcomes.

## Method

### Study design, setting and recruitment

This study was an online cross-sectional survey of mental healthcare staff. The project was conducted at South London and Maudsley NHS Foundation Trust (SLaM), one of the largest unit providers of mental health services in Europe, serving the 1.3 million residents of four boroughs in South London. A healthcare worker was defined as any member of staff that provides care or helps support people with mental health problems or addiction. Healthcare workers, aged >18 years and working at SLaM at the time of the study were eligible to take part. Exclusion criteria included working at SLaM in roles other than healthcare.

Participants were approached via SLaM mailing lists and through social media (e.g. LinkedIn and Twitter). Recruitment posters were also displayed throughout SLaM services and the research team promoted the study through presentations to SLaM employees. The study was also advertised in the King's College London Fortnightly Recruitment Circular. All participants were fully informed of the nature of the study through an information sheet and had to provide informed consent to take part in the study.

### Data collection

The questionnaire was administered with Qualtrics software for Windows (Qualtrics, Provo, Utah, USA; see https://www.qualtrics.com), which could be accessed and completed on a participants’ telephone or laptop. All data collected was anonymised via Qualtrics. Each participant was assigned a random identification number before data analysis. Unique participants were identified via IP addresses to calculate the participation rate. The study was approved by the King's College London Ethics Committee (reference MRSU-21/22-28576). Data was collected from April 2022 until September 2022.

### Measures

Participants answered questions to capture remote working arrangements during the COVID-19 pandemic. They were asked whether they had flexibility or not to work remotely before the COVID-19 pandemic; whether the type of remote work they had to undertake at the time of the pandemic was the same as usual, consisted of different tasks or different scheduling; and how many hours a week on average they worked remotely. Participants were asked to state whether they felt that their levels of productivity, stress and satisfaction, when working remotely during the pandemic, were lower, equal to or higher than that of office work (Supplementary Appendix 1 available at https://doi.org/10.1192/bjo.2024.7). Productivity, stress and satisfaction were all measured by a subjective self-reported response to a single question.

A self-report questionnaire comprising five scales was also administered to all participants, to collect data on their current experiences of loneliness, workplace isolation, perceived social support and well-being. Loneliness was assessed with the UCLA Loneliness Scale.^30^ Workplace isolation was assessed with the Workplace Isolation Scale.^14^ Perceived social support was assessed with the Perceived Social Support Scale.^31^ Well-being was measured on the WHO-5 Well-Being Index,^32^ and the General Health Questionnaire-12 (GHQ-12) was used to measure mental distress.^33^ The UCLA Loneliness Scale shows a high convergent validity, as it was significantly correlated with other measures of loneliness such as the NYU Loneliness Scale.^30^ Marshall and colleagues^14^ also provided evidence of discriminant validity of the Workplace Isolation Scale with Wittenberg and Reis^34^ emotional and social loneliness subscales. The Perceived Social Support Scale has previously been used and validated as a measure of perceived social support in the UK Health and Lifestyle Survey.^35^ The WHO-5 has shown high validity as a measure of depression across a wide range of fields.^36^ The GHQ-12 questionnaire has previously been validated for use with staff in England's NHS, and was found to have a high correlation of 0.70 with the Clinical Interview Schedule – Revised.^37^ The GHQ-12 is thought to be a complementary measure to the Perceived Social Support Scale.^31^

### Statistical analysis

To investigate whether the number of hours worked remotely was associated with a difference in level of productivity, stress and work satisfaction compared with in-person office work, a Pearson's chi-Squared test was used. Descriptive statistics were calculated for each of the scales, and correlations between loneliness, workplace isolation, perceived social support, well-being and mental distress outcomes were investigated with Pearson's correlations (*r*). Regression analyses were performed to investigate whether loneliness, workplace isolation and perceived social support were significant as factors associated with levels of well-being and mental distress. A moderation analyses was conducted, using the PROCESS macro for SPSS (version 28.0 for Windows, IBM Corp, Armonak, New York), to determine whether perceived social support had an influence on the association between loneliness and workplace isolation and well-being. Missing data were omitted on an analysis-by-analysis basis and valid percentages are reported.

## Results

### Sample characteristics

A total of 477 participants completed the survey (14% of 3418 healthcare workers) with an age range of 21–77 years (mean 40.0, s.d. = 12.1). This sample is representative of the socioeconomic characteristics of gender and ethnicity in the population of healthcare workers at SLaM at the time of the study (Table 1).

**Table 1:** Sample demographics

	*n*	% of sample total	SLaM total population %
Gender			
Male	112	21.5	28.8
Female	406	78.1	71.2
Non-binary	2	0.4	
Ethnicity			
White	367	70.6	50.6
Black	61	11.7	31.5
Asian	41	1	7.3
Latino	5	7.9	
Other	33	6.3	8.2
Prefer not to say	13	2.5	2.4
How many hours a week did you work remotely?			
0–5	34	7	
6–14	83	17	
15–30	159	33	
≥31	210	43	

Only a very small proportion (*n* = 15, 3.1%) worked remotely before the pandemic, but the vast majority had spent time working remotely since COVID-19 (*n* = 476, 96.9%). Many participants worked more than 31 h a week remotely during the COVID-19 pandemic (*n* = 209, 43.1%).

### Remote working

Using a Pearson's chi-squared test, the number of hours a week a person worked remotely during the pandemic was investigated with levels of stress, productivity and work satisfaction during this time. The number of hours a person worked remotely during the pandemic was significantly associated with the level of stress they experienced during this time (*χ^2^*(6, 481) = 17.17, *P* < 0.05). Those who had worked more than 31 h a week remotely during the pandemic reported experiencing higher current stress when working remotely than those who worked fewer hours remotely. There was a significant difference in productivity when working remotely and the number of hours worked remotely (*χ^2^*(6, 480) = 17.48, *P* < 0.05). Those who had worked more than 31 h a week remotely were more productive than those who had worked fewer hours a week remotely. There was a significant difference in current work satisfaction when working remotely and the number of hours previously worked remotely (*χ^2^*(6, *480*) = 15.01, *P* = 0.02). Those who had worked more hours remotely per week reported lower work satisfaction when doing so.

### Investigating the association between each predictor variable and well-being

The multiple regression model (Table 2) revealed that, together, loneliness, workplace isolation and perceived social support accounted for 35% of the variance in the WHO-5 Well-Being Index scores (F(3, 397) = 69.46, *P* < 0.001).

**Table 2:** Results of multiple regression analysis for associations between loneliness, workplace isolation and perceived social support (predictors) and well-being, measured on the WHO-5 Well-Being Index (outcome)

WHO-5	*R^2^*	*B*	s.e.	*β*	95% CI for *B*
Model	0.35				
Loneliness		−0.19*	0.02	−0.45	−0.24 to −0.15
Workplace isolation		0.04	0.02	0.09	0.004–0.07
Perceived social support		0.32*	0.09	0.16	0.13–0.50

* *P* < 0.05.

Examining unique associations between each independent variable and well-being, lower well-being scores were associated with higher loneliness scores (B = −0.19, *P* < 0.001), but not with higher workplace isolation scores (B = 0.04, *P* = 0.08). The multiple regression model also found that, adjusting for loneliness and workplace isolation, perceived social support was positively associated with well-being score (B = 0.32, *P* < 0.001).

### Investigating the association between each predictor variable and mental distress

The multiple regression model (Table 3) revealed that, together, 14.0% of the variance in the GHQ-12 scores was explained by the set of independent variables (F(3, 374) = 19.68, *P* < 0.001).

**Table 3:** Results of multiple regression analysis for associations between loneliness, workplace isolation and perceived social support (predictors) and well-being, measured on the General Health Questionnaire-12 (GHQ-12) (outcome)

GHQ-12	*R^2^*	*B*	s.e.	*β*	95% CI for *B*
Model	0.14				
Loneliness		0.16*	0.04	0.28	0.09–0.24
Workplace isolation		−0.08*	0.03	−0.15	−0.14 to 0.02
Perceived social support		0.07	0.15	−0.02	−0.23 to 0.37

* *P* < 0.05.

Examining unique associations with each independent variable, GHQ-12 scores were positively associated with loneliness (B = 0.16, *P* = 0.04). Multiple regression analysis also revealed that higher workplace isolation was associated with GHQ-12 scores (B = −0.08, *P* = 0.03). However, level of perceived social support was not associated with GHQ-12 scores (B = 0.07, *P* = 0.15).

### Secondary analysis

A moderation analysis was conducted to investigate the potential moderating role of perceived social support on the association between loneliness and well-being, and between workplace isolation and well-being. There was no significant interaction between loneliness, perceived social support and well-being (B = −0.0006, *P* = 0.94), nor between workplace isolation, perceived social support and well-being (B = 0.08, *P* = 0.19).

A moderation analysis was also conducted to investigate the potential moderating role of perceived social support on the association between loneliness and mental distress, and between workplace isolation and mental distress. There was no significant interaction between loneliness, perceived social support and mental distress (B = −0.02, *P* = 0.10), nor between workplace isolation, perceived social support and well-being (B = −0.009, *P* = 0.37).

## Discussion

### Key findings

As expected, there was a large increase in the proportion of healthcare staff who worked remotely, from 3.1% before the pandemic to 96.9%, with most respondents reporting that they had worked over 31 h a week remotely. The reported hours per week worked remotely were associated with perceived changes in productivity, stress and work satisfaction during this time. Those who had worked more hours remotely during the pandemic reported higher stress and lower workplace satisfaction, but had also felt more productive compared with their previous office work arrangements. In terms of the current well-being of healthcare workers, half of the staff members surveyed reported moderate or greater degrees of loneliness. The average GHQ-12 scores of 16.5 suggested significant mental distress, with scores of 12 or higher typically being considered as a clinical case. Those experiencing greater loneliness and workplace isolation at the time of the study also reported poorer well-being. We found no evidence of a moderating effect of perceived social support on the associations between well-being and loneliness or workplace isolation, respectively.

### Comparison with previous literature

Previous literature reports that around 50% of UK employees have worked remotely at some point since the pandemic, compared with our sample of 96.9%.^38^ This may be explained by the recruitment strategy used aiming to explore remote working, which might have encouraged those with experience in working remotely to respond. Although many of our self-selected respondents worked remotely for more than 31 h a week during the COVID-19 pandemic, we do not know whether this sample worked exclusively remotely, so this could not be explored further.

The finding that people who spent more hours a week working remotely felt more productive may be because of more freedom in work or the use of online platforms promoting productivity.^19^ However, those who reported having worked more hours remotely at some point since the pandemic, also reported higher levels of stress and lower work satisfaction at that time. This could be attributable to healthcare workers having to navigate prioritisation of care and compromises in ability to perform certain aspects of their roles.^39^

There is substantial evidence highlighting the need to consider the well-being of healthcare workers during the COVID-19 pandemic.^40^ However, UK guidelines on this topic need further investigation at contextual characteristics that could be affecting the success of these, as they do not appear to be effective.^41^ Our findings can be read in the context of work indicating that 38% of NHS employees who had to work from home since the COVID-19 pandemic did not feel that they had sufficient resources to do so, and 44% felt their contribution had not been acknowledged.^42^ This raises the possibility of strategies to mitigate the effects of high workplace isolation on well-being.

Perceived social support was associated with better contemporaneous well-being. Promoting perceived social support amongst healthcare workers in the NHS may allow for improvements in well-being of the employees and for the organisation's delivery of care.^43^ The results underpin how social support can provide benefits in relation to the well-being of healthcare workers during periods of disaster.^44^ However, in this study, factors not taken into consideration include financial difficulties during this crisis or uncertainty and fear of the future.^27^ A combination of these individual factors may weaken the predictive power of the interaction of loneliness or workplace isolation and perceived social support to well-being, and hence need to be taken into consideration.

### Strengths and limitations

This study reports findings from a large mental healthcare provider, adding to the limited literature on the impact of the COVID-19 pandemic and remote working on healthcare workers in the UK and providing further understanding of how this affected NHS employees. However, the response rate of 14% of SLaM healthcare staff limits the generalisability of the results to the entire population. The cross-sectional survey nature of this study and data collection methods prevents the ability to test causative effects of remote working. Additionally, because of the sample size, more complex models such as a mediated moderation analysis were not possible to conduct. Although hours of remote working per week was captured, the period that this lasted for and whether they were working remotely at the time of data collection was not recorded, and hence the full scope of current working arrangements was not explored. Furthermore, the self-report nature of online surveys can lead to response bias being conveyed in the results, because of social desirability.^45^ We were also unable to account for the impact of changes to social life outside of work, which may have had an effect on the outcome measures. Research has also suggested that those who are high in loneliness, as evident in this sample, are more likely to report less accurately on self-report measures because of poor introspection and self-evaluation abilities.^46^ However, the measures used in the study have a high validity in measuring the outcomes.

### Implications of these findings for future practice and research

The low well-being and high GHQ-12 scores reported in this study call for further investigation into the current well-being of healthcare workers in the UK. This is imperative because of the importance of the work conducted by healthcare staff and the high-quality patient care that they are required to deliver, and the growing trend for some roles to be conducted exclusively or partially online. Several practical implications arise, including the need to study hybrid working practices. Poor well-being of healthcare employees is closely linked to patient errors, which cost the NHS £3.3 billion each year.^47^ Interventions should be developed to increase perceived social support and promote more positive well-being of NHS healthcare workers. The context and conditions that healthcare workers face during such times needs also to be considered, and a longitudinal study could investigate this and provide direction for change.

Other reasons for current poor well-being, aside from the shift to remote working, need to be considered. The NHS has been placed under immense pressure since the COVID-19 pandemic, and at the time of conducting this survey, the SLaM was under operational pressure escalation level 4 (OPEL 4), possibly influencing the findings obtained. This level of pressure in the NHS is the most catastrophic, and is described as a state of escalation that leaves the trusts unable to deliver comprehensive care, and there is increased potential for patient care and safety to be compromised.^48^ This also provides a strong rational for the need for research into healthcare employees at SLaM at this time.

In conclusion, since the COVID-19 pandemic NHS healthcare employees have seen a major shift to remote working, with little prior evidence on the impact this could have on stress, productivity and work satisfaction. This study has pointed toward cross-associations between loneliness and workplace isolation and poor well-being. NHS trusts should investigate ways to promote social support for staff, which in turn should improve healthcare employee's well-being and care provision. Future research on the impact of remote working on healthcare workers should take in to account other potential difficulties staff are experiencing with work–life balance, and explore what type of working arrangements employees prefer. In the NHS, identifying and understanding the potential positive and negative effects of remote working and its influence on healthcare workers will be vital in protecting their well-being and identifying possible interventions in potential future pandemics in the UK.

## Supporting information

O'Hare et al. supplementary material 1 O'Hare et al. supplementary material

O'Hare et al. supplementary material 2 O'Hare et al. supplementary material

O'Hare et al. supplementary material 3 O'Hare et al. supplementary material

O'Hare et al. supplementary material 4 O'Hare et al. supplementary material

## Data Availability

The data that support the findings of this study are available from the corresponding author, M.P.d.C., upon reasonable request.
