# Supplementary material for: A cross-sectional investigation on remote working, loneliness, workplace isolation, well-being and perceived social support in healthcare workers
Source: BJPsych Open. 2024 Feb 26;10(2):e50. doi: 10.1192/bjo.2024.7 (PMC10897687; doi:10.1192/bjo.2024.7)
Supplement: O'Hare et al. supplementary material 1 — O'Hare et al. supplementary material [file S2056472424000073sup001.docx]

**Title: Appendix A**

***Description: Questionnaire***

*Demographics*

Age

Gender

Job role within SLAM

*Remote Working Arrangements of the Sample*

1. Before COVID-19 did you only work remotely? Yes/No

2. Before COVID-19 did your job allow you flexibility to work remotely? Yes/No

3. Since COVID-19 have you had to work remotely at some point? Yes/No

*Characteristics and quality of working from home ^38^*

Was the type of remote work

- Same as office work

- Different tasks

- Different schedule

- Different salary

How many hours a week did you work?

<36 or ≥36

Was your productivity lower, equal to or higher than office work?

Was your stress lower, higher or equal to that of office work?

Was your work Satisfaction lower, equal to or higher than office work?
