# Supplementary material for: A cross-sectional investigation on remote working, loneliness, workplace isolation, well-being and perceived social support in healthcare workers
Source: BJPsych Open. 2024 Feb 26;10(2):e50. doi: 10.1192/bjo.2024.7 (PMC10897687; doi:10.1192/bjo.2024.7)
Supplement: O'Hare et al. supplementary material 2 — O'Hare et al. supplementary material [file S2056472424000073sup002.docx]

**Title: Appendix B**

***Description: Remote working characteristics of the sample***

| **Question** | | **N** | **Percentage (%)** | | | |
| --- | --- | --- | --- | --- | --- | --- |
| Before COVID-19 did you only work remotely? | |  | | |  | |
| *Yes* | | 15 | | | 3.1 | |
| *No* | | 476 | | | 96.9 | |
| Before COVID-19 did your job allow you flexibility to work remotely? | |  | | |  | |
| *Yes* | | 91 | | | 18.7 | |
| *No* | | 399 | | | 81.3 | |
| Since COVID-19 have you had to work remotely at some point? | |  | | |  | |
| *Yes* | | 476 | | | 96.9 | |
| *No* | | 15 | | | 3.1 | |
| Was the type of work… | |  | | |  | |
| *The same as usual* | | 396 | | | 82.2 | |
| *Different tasks* | | 53 | | | 11.0 | |
| *Different schedule*  *Different salary* | | 17  1 | | | 3.5  0.2 | |
| *Other* | | 15 | | | 3.1 | |
| How many hours a week did you work remotely? | |  | | |  | |
| *0 – 5* | | 34 | | | 7.0 | |
| *6 – 16* | | 83 | | | 17.1 | |
| *15 – 30* | | 159 | | | 32.8 | |
| *31 or more* | | 209 | | | 43.1 | |
| Was your productivity higher, equal to or lower than office work? | |  | | |  | |
| *Higher* | | 226 | | | 47.0 | |
| *Equal* | | 166 | | | 34.5 | |
| *Lower* | | 89 | | | 18.5 | |
| Was your stress higher, equal to or lower than office work? | |  | | |  | |
| *Higher* | | 168 | | | 34.8 | |
| *Equal* | | 119 | | | 24.6 | |
| *Lower* | | 196 | | | 40.6 | |
| Was your satisfaction higher, equal to or lower than office work? | |  | | |  | |
| *Higher* | | 143 | | | 29.7 | |
| *Equal* | | 169 | | | 35.1 | |
| *Lower* | | 170 | | | 35.3 | |
