# Supplementary material for: A cross-sectional investigation on remote working, loneliness, workplace isolation, well-being and perceived social support in healthcare workers
Source: BJPsych Open. 2024 Feb 26;10(2):e50. doi: 10.1192/bjo.2024.7 (PMC10897687; doi:10.1192/bjo.2024.7)
Supplement: O'Hare et al. supplementary material 3 — O'Hare et al. supplementary material [file S2056472424000073sup003.docx]

Title : Appendix C

***Measures table***

| Variable | Measure | Source | Definition |
| --- | --- | --- | --- |
|  |  |  |  |
| Loneliness | The UCLA Loneliness Scale | Russell, 1996 | Loneliness is a subjective feeling of prolonged emotional distress based on dissonance between perceived and desired social relations |
|  |  |  |  |
| Workplace Isolation | Workplace Isolation Measure | Marshall et al., 2007 | Workplace isolation is an individual’s perceived isolation from their colleagues and from the organisation's support network |
| Perceived Social Support | The Perceived Social Support Score | Shields & Price, 2005 | Perceived social support refers to how sufficient and available one perceives their social support to be |
|  |  |  |  |
| Wellbeing | The GHQ-12 | Goldberg, 1978 |  |
|  | WHO-5 Wellbeing Scale | World Health Organisation, 1998 | Health is a state of complete physical, mental, and social well-being and not merely the absence of disease or infirmity |
| Stress | Self-reported | World Health Organisation, 2023 | Stress can be defined as a state of worry or mental tension caused by a difficult situation. Stress is a natural human response that prompts us to address challenges and threats in our lives. |
| Productivity | Self-reported | Oxford English Dictionary | Productivity is defined as the effectiveness of productive effort |
| Work Satisfaction | Self-reported | Locke, 1969 | Work satisfaction is a pleasurable emotional state that a person attributes to their role facilitating the achievement of job values. |
